# Sulforaphane Suppresses H_2_O_2_-Induced Oxidative Stress and Apoptosis via the Activation of AMPK/NFE2L2 Signaling Pathway in Goat Mammary Epithelial Cells

**DOI:** 10.3390/ijms24021070

**Published:** 2023-01-05

**Authors:** Dan Shao, Zhen Gao, Ying Zhao, Mingzhen Fan, Xiaoe Zhao, Qiang Wei, Menghao Pan, Baohua Ma

**Affiliations:** 1 Department of Clinical Veterinary Medicine, College of Veterinary Medicine, Northwest A&F University, Yangling 712100, China; 2 Key Laboratory of Animal Biotechnology of the Ministry of Agriculture, Northwest A&F University, Yangling 712100, China

**Keywords:** apoptosis, oxidative stress, sulforaphane, goat mammary epithelial cells, AMPK/NFE2L2

## Abstract

Oxidative stress in high-yielding dairy goats adversely affects lactation length, milk quality, and the economics of dairy products. During the lactation period, goat mammary epithelial cells (GMECs) are often in a state of disordered metabolic homeostasis primarily caused by the overproduction of reactive oxygen species (ROS). Sulforaphane (SFN), an electrophilic compound that is enriched in broccoli, is a promising antioxidant agent for future potential clinical applications. The objective of the present study was to investigate the function of SFN on hydrogen peroxide (H_2_O_2_)-induced oxidative damage in primary GMECs and the underlying molecular mechanisms. Isolated GMECs in triplicate were pretreated with SFN (1.25, 2.5, and 5 μM) for 24 h in the absence or presence of H_2_O_2_ (400 μM) for 24 h. The results showed that SFN effectively enhanced superoxide dismutase (SOD) activity, elevated the ratio of glutathione (GSH)/glutathione oxidized (GSSG), and reduced H_2_O_2_-induced ROS and malondialdehyde (MDA) production and cell apoptosis. Mechanically, SFN-induced nuclear factor erythroid 2-related factor 2 (NRF2/NFE2L2) translocation to the nucleus through the activation of the adenosine monophosphate-activated protein kinase (AMPK) signaling pathway coupled with inhibition of the caspase apoptotic pathway. In addition, GMECs were transfected with NFE2L2 small interfering RNA (NFE2L2 siRNA) for 48 h and/or treated with SFN (5 μM) for 24 h before being exposed to H_2_O_2_ (400 μM) for 24 h. We found that knockdown of NFE2L2 by siRNA abrogated the preventive effect of SFN on H_2_O_2_-induced ROS overproduction and apoptosis. Taken together, sulforaphane suppressed H_2_O_2_-induced oxidative stress and apoptosis via the activation of the AMPK/NFE2L2 signaling pathway in primary GMECs.

## 1. Introduction

Dairy product safety is a growing public health concern worldwide. The health of dairy goats is of considerable relevance to the safety, quality, and health-promoting attributes of dairy products. Physiological changes in lactating dairy goats, particularly high-yielding dairy goats, determine that the organism is often in a state of metabolic homeostasis disorder and result in oxidative stress [1,2,3]. A prolonged state of oxidative stress not only affects the intensive growth of the mammary gland but also supports the appearance of mastitis, which causes a reduction in milk yield and unfavorable changes in milk composition [4,5]. For these reasons, better antioxidant strategies are of great significance to ensure the healthy growth and lactation of dairy goats and to produce high-quality milk.

Oxidative stress plays a vital role in many physiological and pathological processes [6]. Reactive oxygen species (ROS) are natural byproducts of redox metabolism, but ROS overproduction leads to oxidative stress and cellular damage [7]. Goat mammary epithelial cells (GMECs), primary components of the mammary gland for milk synthesis during lactation, undergo intensive metabolism and produce a large amount of ROS [8,9,10]. In such a situation, GMECs are very sensitive to oxidative stress [11]. It is reported that oxidative stress participates in regulating gene expression and apoptosis, eventually leading to cell injury [12]. Altogether, antioxidant drugs should be developed in order to prevent excessive ROS production while alleviating cell apoptosis.

Nuclear factor erythroid 2-related factor 2 (NRF2/NFE2L2) is a pivotal transcription factor that regulates cellular redox homeostasis [13]. It regulates the expression of numerous cytoprotective proteins associated with detoxification and antioxidant responses, including heme oxygenase 1 (HMOX1), quinone oxidoreductase 1 (NQO1), glutamate–cysteine ligase catalytic (GCLC), glutamate–cysteine ligase modifier subunit (GCLM), and other antioxidant proteins by binding to antioxidant response elements (AREs) [14,15]. Previous research has shown that activation of the NFE2L2 pathway contributes to cellular defense against H_2_O_2_-induced oxidative damage in bovine MECs [8,16]. Therefore, the NFE2L2 activation has been a target for the development of promising therapeutic strategies against oxidative stress in goat mammary epithelial cells.

Sulforaphane (SFN), an electrophilic compound, is isolated from cruciferous vegetables [17]. It is pleiotropic and possesses antioxidative and anti-inflammatory potentiality [18]. It has been reported that AMP-activated protein kinase (AMPK) activation is a potential therapeutic target against oxidative-stress-induced diseases [19]. Given the versatility of SFN and the critical role of AMPK in the regulation of NFE2L2 and oxidative stress, we hypothesized that SFN activates the AMPK/NFE2L2 pathway to prevent H_2_O_2_-induced oxidative damage in GMECs. In this study, compound C (CC) and NFE2L2 siRNA (si-NFE2L2) were used to inhibit AMPK activation and downregulate NFE2L2 abundance in primary GMECs, respectively. The objective of the study was to investigate the effect and mechanism of SFN on H_2_O_2_-induced oxidative stress and cell apoptosis in GMECs.

## 2. Results

### 2.1. SFN Enhanced the Antioxidant Capacity in H_2_O_2_-Induced GMECs

**Figure 1:**
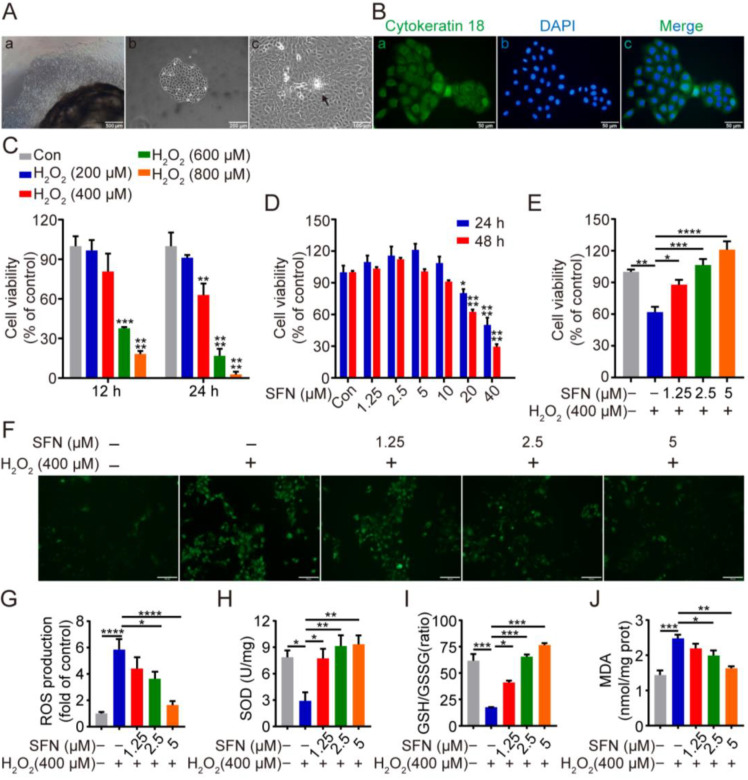
Sulforaphane (SFN) enhanced the antioxidant capacity in primary goat mammary epithelial cells (GMECs). Cells were treated with SFN (1.25, 2.5, 5 μM) for 24 h before exposed to H_2_O_2_ (400 μM) for 24 h. (**Aa**) Primary goat mammary epithelial cells (GMECs) moved out of tissue block after culture for 8 days. Scale bars: 500 μm. (**Ab**) GMECs for passage 1. Scale bars: 200 μm. (**Ac**) Dome-like structures of GMECs (arrow). Scale bars: 100 μm (**B**) Immunostaining analysis of cytokeratin 18 (CK18) (**Ba**) and nuclear staining with DAPI (**Bb**) in GMECs with merge image (**Bc**). Scale bars: 50 μm. (**C**) Cell viability of GMECs after treatment with different concentrations of H_2_O_2_ (0~800 μM) for 12 h or 24 h. One-way ANOVA, Dunnett’s post hoc test. (**D**) Cell viability of GMECs after treatment with different concentrations of SFN (0~40 μM) for 24 h or 48 h. One-way ANOVA, Tukey’s post hoc test. (**E**) Cell viability of GMECs after treatment with different concentrations of SFN (1.25, 2.5, and 5 μM) for 24 h in the absence or presence of H_2_O_2_ (400 μM) for 24 h. (**F**) Reactive oxygen species (ROS) production was detected by the fluorescence intensity of 2,7-Dichlorodihydrofluorescein diacetate (DCFH-DA) using fluorescent microscope. Scale bars: 50 μm. (**G**) Quantification of ROS production. (**H**–**J**) Superoxide dismutase (SOD) activity, glutathione (GSH)/glutathione oxidized (GSSG) ratio, and malondialdehyde (MDA) production were detected in different groups. One-way ANOVA, Tukey’s post hoc test. Data are the mean ± SEM (*n* = 3). * *p* < 0.05, ** *p* < 0.01, *** *p* < 0.001, and **** *p* < 0.0001.

As shown in Figure 1A, isolated cells displayed a cobblestone-like shape and colony formation, which are typical epithelial cell morphology. The positive immunofluorescent staining for luminal epithelial cells marker cytokeratin-18 (CK18) suggested that the isolated cells were GMECs (Figure 1B). To investigate the cytotoxicity of SFN or/and H_2_O_2_ on GMECs, cell viability was detected after treatment with different concentrations of SFN or/and H_2_O_2_ for the indicated time. SFN at 1.25, 2.5, and 5 μM promoted cell proliferation without cytotoxicity in a time- and dose-dependent manner (Figure 1D), and H_2_O_2_ (400, 600, and 800 μM) significantly reduced the viability of GMECs at 24 h (Figure 1C). Meanwhile, SFN attenuated the decreased viability of GMECs caused by H_2_O_2_ in a dose-dependent manner (Figure 1E). According to the results, concentrations of 1.25, 2.5, and 5 μM of SFN were chosen as the experimental conditions, and 400 μM H_2_O_2_-induced cells for 24 h were selected for further experiments.

To investigate the potential antioxidative capacity of SFN, the effect of SFN on ROS level, SOD activity, MDA activity, and GSH/GSSH ratio in GMECs were measured. As shown in Figure 1F,G,J, H_2_O_2_ treatment resulted in high levels of ROS and MDA, and pretreatment with SFN remarkably suppressed the level of these at a concentration of 2.5 μM and 5 μM. Meanwhile, treatment with H_2_O_2_ reduced the activity of SOD and the ratio of GSH/GSSG, whereas pretreatment of SFN effectively rescued the decline in a dose-dependent manner (Figure 1H,I). Taken together, the enhancement of SOD activity and the elevation in the GSH/GSSG ratio suggest that SFN enhances the antioxidant ability of GMECs, which reduces H_2_O_2_-induced ROS and MDA generation.

### 2.2. SFN Inhibited H_2_O_2_-Induced Cell Apoptosis

**Figure 2:**
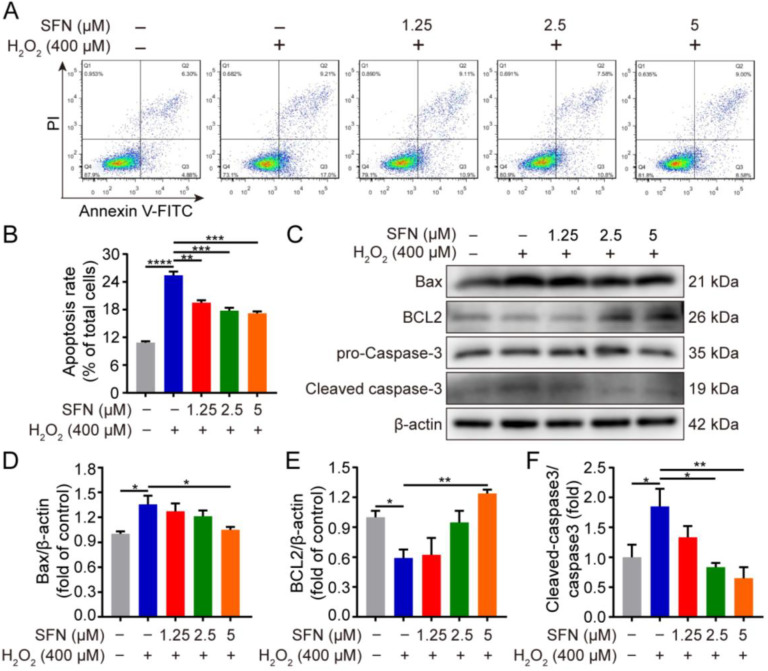
SFN suppresses H_2_O_2_-induced apoptosis. GMECs were treated with SFN for 24 h following H_2_O_2_ treatment. (**A**) Detection of apoptosis using annexin V-FITC and PI staining assay through flow cytometry. (**B**) Statistical analysis of the apoptotic rate. (**C**) Western blot analysis of recombinant BCL2 associated X protein (Bax), B cell leukemia/lymphoma 2 (BCL2), procaspase-3, and cleaved caspase-3 protein expression. (**D**) Quantitative analysis of Bax protein level. (**E**) Quantitative analysis of BCL2 protein level. (**F**) Quantitative analysis of cleaved caspase-3/procaspase-3 protein level. One-way ANOVA, Tukey’s post hoc test. Data are the mean ± SEM (*n* = 3). * *p* < 0.05, ** *p* < 0.01, *** *p* < 0.001, and **** *p* < 0.0001.

To evaluate the effect of SFN on cell apoptosis, flow cytometry and Western blotting were conducted. As shown in Figure 2A,B, flow cytometry showed that SFN pretreatment significantly decreased the rate of apoptosis compared with H_2_O_2_-treated cells. Furthermore, H_2_O_2_ treatment upregulated the expression of Bax with a concomitant inhibition of BCL2 protein expression (Figure 2C–E). In contrast, SFN pretreatment significantly attenuated the changes caused by H_2_O_2_ (Figure 2C–E). Similarly, SFN pretreatment resulted in reduced cleaved caspase-3 activity and had no effect on the abundance of procaspase-3, leading to a decrease in the ratio of cleaved caspase-3/procaspase-3 compared with H_2_O_2_ treatment (Figure 2C,F). These data indicate that SFN has an antiapoptotic effect on H_2_O_2_-induced cells.

### 2.3. SFN Accelerated AMPK Phosphorylation, Promoted NFE2L2 Nuclear Translocation, and Upregulated Antioxidant Enzyme Expression in Primary GMECs

**Figure 3:**
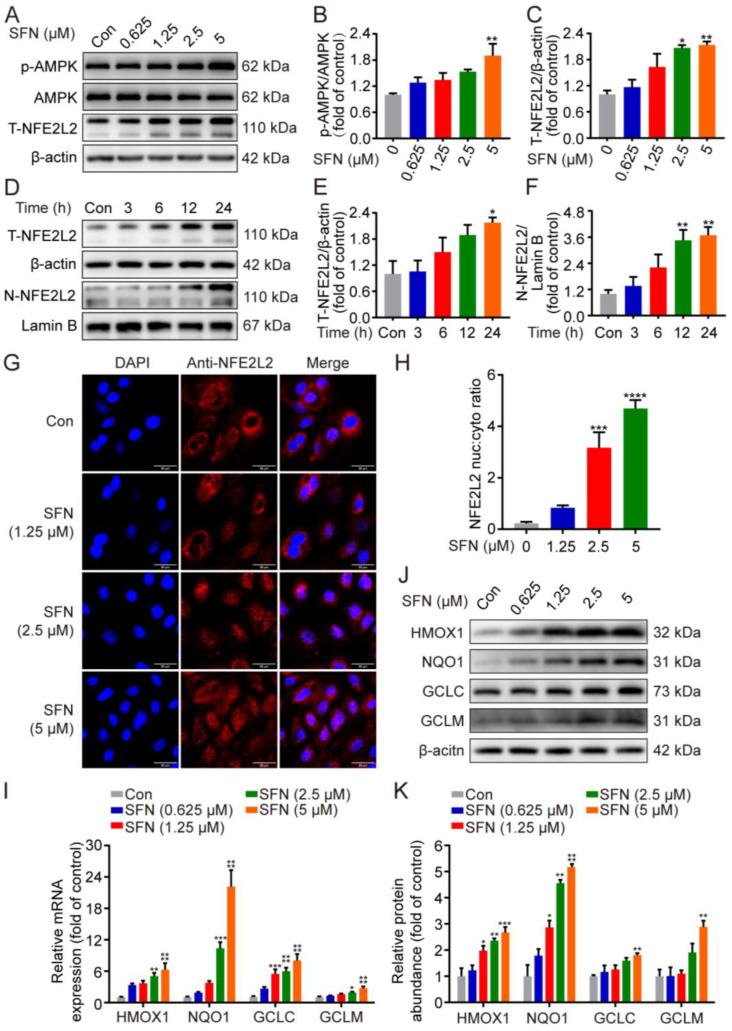
SFN accelerated AMPK phosphorylation, promoted NFE2L2 nuclear translocation, and upregulated antioxidant enzyme expression in primary GMECs. (**A**) Phosphorylated (p)-AMPK and total NFE2L2 (T-NFE2L2) protein levels in total cell lysates were determined by Western blot from SFN-treated GMECs for the indicated dose. (**D**) NFE2L2 protein expression in total cell lysates and nuclear was detected using Western blot analysis from SFN-treated cells for the indicated times. (**B**,**C**,**E**,**F**) Quantitative analysis of p-AMPK (different dose), T-NFE2L2 (different dose), T-NFE2L2 (different time), and N-NFE2L2 (different time) protein levels. One-way ANOVA, Dunnett’s post hoc test. (**G**) Immunofluorescence images of NFE2L2 in GMECs treated with SFN for 24 h. NFE2L2 and the nuclei were labeled with Alexa Fluor 594 (red) and DAPI (blue), respectively. Scale bars: 50 μm. (**H**) Quantification of results shown in (**G**). *n* = 30 randomly selected cells. (**I**) Relative mRNA expression of heme oxygenase 1 (HMOX1), NADH quinone oxidoreductase 1 (NQO1), glutamate–cysteine ligase catalytic subunit (GCLC), and glutamyl–cysteine ligase modulatory subunit (GCLM) was analyzed by quantitative real-time PCR (qRT-PCR) from SFN-treated GMECs for the indicated times. Two-way ANOVA, Dunnett’s post hoc test. (**J**) HMOX1, NQO1, GCLC, and GCLM protein levels in total cell lysates were determined by Western blot from SFN-treated GMECs for the indicated dose. (**K**) Quantitative analysis of HMOX1, NQO1, GCLC, and GCLM protein levels. Two-way ANOVA, Dunnett’s post hoc test. Data are the mean ± SEM (*n* = 3). * *p* < 0.05, ** *p* < 0.01, *** *p* < 0.001, and **** *p* < 0.0001.

In order to confirm whether SFN exerts its protective effects by activating the NFE2L2 signaling pathway, the expression of NFE2L2 was first determined in GMECs. As depicted in Figure 3A–C, treatment with SFN prominently upregulated the phosphorylation of AMPK at 5 μM and significantly elevated NFE2L2 protein expression in a dose-dependent manner. To discover additional NFE2L2 nuclear translocation, Western blot and immunofluorescence experiments were conducted. As shown in Figure 3D–F, the immunofluorescence results showed that NFE2L2 proteins predominantly accumulated in the nucleus after SFN treatment dose-dependently (Figure 3G). Next, qRT-PCR was performed to determine the effect of SFN on the expression of NFE2L2-related genes (*HMOX1*, *NQO1*, *GCLC*, and *GCLM*). Treatment of the GMECs with SFN (1.25, 2.5, and 5 μM) induced a dose-dependent increase in the mRNA level of NFE2L2 targets (Figure 3H). Furthermore, increased transcription was accompanied by upregulated protein expression following SFN treatment (Figure 3I,J). These results suggest that treatment with SFN accelerates AMPK phosphorylation, leads to nuclear translocation of NFE2L2, and increases the expression of its downstream targets.

### 2.4. SFN Activated the AMPK/NFE2L2 Signaling Pathway in H_2_O_2_-Induced GMECs

**Figure 4:**
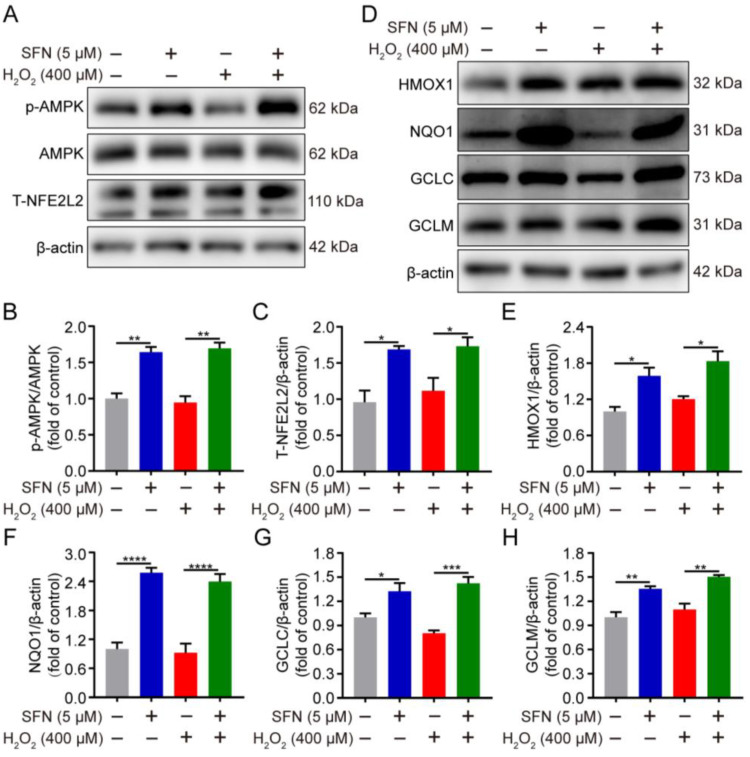
SFN activated the AMPK/NFE2L2 signaling pathway in H_2_O_2_-induced GMECs. Cells were treated with SFN (5 μM) for 24 h before exposed to H_2_O_2_ (400 μM) for 24 h. (**A**) p-AMPK and NFE2L2 protein levels in total cell lysates were determined by Western blot. (**D**) HMOX1, NQO1, GCLC, and GCLM protein levels in total cell lysates were determined by Western blot from SFN-treated GMECs under oxidative stress. (**B**,**C**,**E**–**H**) Quantitative analysis of p-AMPK, T-NFE2L2, HMOX1, NQO1, GCLC, and GCLM protein levels. One-way ANOVA, Tukey’s post hoc test. Data are the mean ± SEM (*n* = 3). * *p* < 0.05, ** *p* < 0.01, *** *p* < 0.001, and **** *p* < 0.0001.

Having shown that phosphorylation of AMPK promotes nuclear accumulation of NFE2L2, regulating ARE-driven gene transactivation [20], we thus speculated whether SFN activates the AMPK/NFE2L2 signaling pathway in H_2_O_2_-induced oxidative stress. The impact of SFN on NFE2L2 and its target proteins under oxidative stress was examined. As shown in Figure 4A,C–H, SFN pretreatment induced higher expression of NFE2L2 and its target proteins compared with that of the H_2_O_2_ treatment. In addition, compared with the H_2_O_2_ group, SFN observably elevated the phosphorylation of AMPK under oxidative stress (Figure 4A,B). Together, the data indicate that SFN activates the AMPK/NFE2L2 signaling pathway in H_2_O_2_-induced GMECs.

### 2.5. SFN Exerted Antioxidant Effects through the AMPK/NFE2L2 Pathway in H_2_O_2_-Induced GMECs

**Figure 5:**
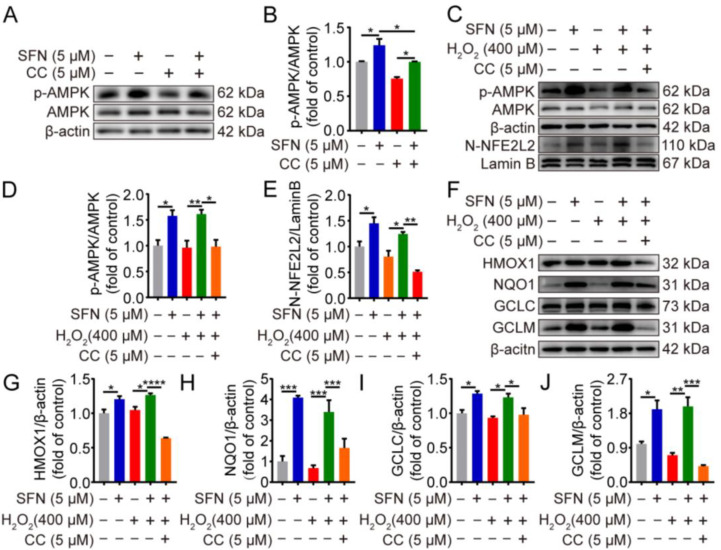
The antioxidant effects of SFN are mediated through AMPK/NFE2L2 pathway in H_2_O_2_-induced GMECs. GMECs were treated with compound C (5 μM) for 4 h in advance and/or were treated with SFN (5 μM) for 24 h before exposed to H_2_O_2_ (400 μM) for 24 h. (**A**) AMPK phosphorylation levels in SFN-treated cells were detected using Western blot analysis. (**C**) p-AMPK, NFE2L2 protein expression levels were determined by Western blot from SFN-treated cells supplementation with compound C under oxidative stress. (**F**) HMOX1, NQO1, GCLC, and GCLM protein levels were detected by Western blot from SFN-treated cell supplementation with compound C under oxidative stress. (**B**,**D**,**E**,**G**–**J**) Quantitative analysis of p-AMPK, NFE2L2, HMOX1, NQO1, GCLC, and GCLM protein levels. One-way ANOVA, Tukey’s post hoc test. Data are the mean ± SEM (*n* = 3). * *p* < 0.05, ** *p* < 0.01, *** *p* < 0.001, and **** *p* < 0.0001.

To test the role of AMPK in SFN-induced NFE2L2 nuclear accumulation, we pretreated GMECs with compound C (CC), an AMPK inhibitor. The results showed that supplementation with compound C decreased the phosphorylation of AMPK in SFN-treated GMECs (Figure 5A,B). Compared with the SFN + H_2_O_2_ group, the combined treatment of SFN and compound C downregulated AMPK activity as well as reduced nuclear accumulation of NFE2L2 under H_2_O_2_-induced oxidative stress (Figure 5C–E). Additionally, AMPK inhibition reverted SFN-induced protein upregulation of HMOX1, NQO1, GCLC, and GCLM after being exposed to H_2_O_2_ (Figure 5F,G). Collectively, these results suggest that AMPK activation is responsible for the antioxidant effect of SFN in H_2_O_2_-induced GMECs.

### 2.6. NFE2L2 Is Required for the Inhibitory Effects of SFN on H_2_O_2_-Induced Oxidative Damage

**Figure 6:**
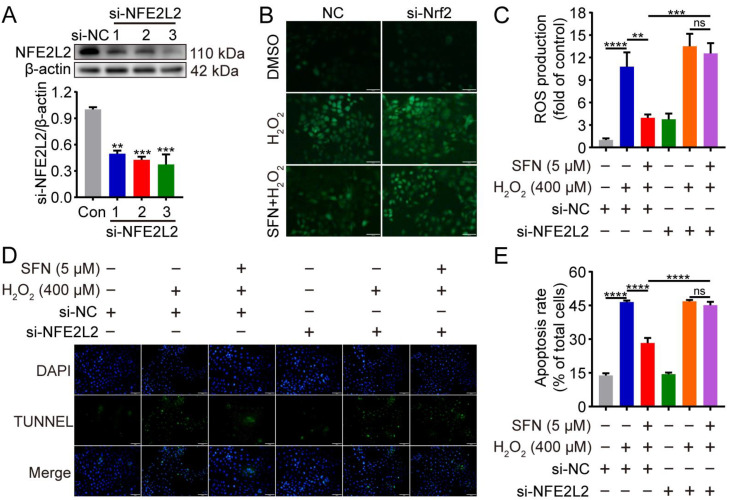
Knockdown of NFE2L2 attenuated the inhibitory effects of SFN on H_2_O_2_-induced oxidative damage. GMECs were transfected with NFE2L2 small interfering RNA (siRNA) for 48 h and/or treated with SFN (5 μM) for 24 h before exposed to H_2_O_2_ (400 μM) for 24 h. (**A**) NFE2L2 protein expression levels in total cell lysates were detected using Western blot analysis. (**B**) ROS production was detected by the fluorescence intensity of DCFH-DA using fluorescent microscope. Scale bar: 100 μm. (**C**) Quantitative analysis of ROS production. (**D**) Cell apoptosis was analyzed by TUNEL staining (TUNEL: green, DAPI: blue). Scale bar: 100 μm. (**E**) Quantification of TUNEL-positive cells (apoptotic cells). One-way ANOVA, Tukey’s post hoc test. Data are the mean ± SEM (*n* = 3). ns: *p* > 0.05, ** *p* < 0.01, *** *p* < 0.001, and **** *p* < 0.0001.

To verify whether the antioxidant activity of SFN was mediated through NFE2L2, we used NFE2L2-silenced GMECs to determine whether SFN pretreatment inhibits ROS production and cell apoptosis upon H_2_O_2_ stimulation. The RNAi efficiency was detected by Western blot experiment. As shown in Figure 6A, transfection with NFE2L2 siRNA for 48 h decreased NFE2L2 expression in GMECs, especially si-NFE2L2-3. Therefore, NFE2L2-siRNA-3 was chosen for subsequent experiments. In the control siRNA condition, pretreatment with SFN suppressed ROS production upon H_2_O_2_ stimulation, but not in conditions without NFE2L2 (Figure 6B,C). This is consistent with the phenotype of cell apoptosis observed by TUNEL and further supports a role for NFE2L2 in the enhancement of antioxidant and anti-apoptosis capacities with SFN (Figure 6D,E). These data indicate that the SFN-induced suppression of ROS production and cell apoptosis are both NFE2L2 dependent.

## 3. Discussion

In the present study, the results demonstrate that SFN plays an important role in suppressing H_2_O_2_-induced oxidative stress and apoptosis in primary GMECs, which is related to the activation of the AMPK/NFE2L2 pathway and inactivation of the caspase-dependent pathway of apoptosis. This led us to speculate that these responses in vitro could have implications for the in vivo application of SFN, especially when the mammary gland undergoes metabolic changes during the periparturient period. Therefore, SFN may be a promising therapeutic agent to mitigate oxidative stress in high-yielding dairy animals.

MECs of ruminants have been prevalently used to study milk synthesis, inflammatory response, and lipid metabolism in vitro [21]. Hydrogen peroxide (H_2_O_2_), as a strong oxidant, has been widely used to induce cellular oxidative stress models [22]. To better simulate oxidative stress in mammary glands, dairy goat primary mammary epithelial cells were treated with H_2_O_2_. In the current study, oxidative stress elicited by H_2_O_2_ causes an increase in ROS and MDA production, leading to lipid peroxidation and a compromised antioxidant system, which promotes further oxidative damage of GMECs. Conversely, sulforaphane is a powerful antioxidant due to its ability to scavenge ROS directly and enhance the activity of endogenous antioxidant enzymes. The enhanced SOD activity and GSH/GSSG ratio proved that SFN pretreatment strengthens the antioxidant system in H_2_O_2_-induced GMECs, and was also confirmed by a decrease in ROS and MDA production. However, as with all in vitro studies of isolated MECs, it is unclear whether the oxidative stress model responds similarly to in vivo conditions experienced by high-yielding dairy goats. Therefore, to address the in vivo relevance of our findings, the clinical study of SFN should be conducted to determine its role in redox regulation.

Many studies about the possible role of NFE2L2, including its targets, have emerged in recent years. Focusing on the NFE2L2 pathway is a practical tactic to ameliorate oxidative stress. Ma et al. found that reducing nuclear translocation and transcriptional activity of NFE2L2 is sensitive to oxidative stress, causing oxidative damage to bovine MECs [16]. Previous research has shown that SFN is an activator of NFE2L2 and upregulates antioxidant enzyme expression, mediating cellular defense against oxidative stress [23,24]. However, the function of SFN and its modulation of NFE2L2 has not been investigated in GMECs. In this study, SFN dramatically activated NFE2L2 in a time-dependent and dose-dependent manner. Moreover, the mRNA and protein expression of HMOX1, GCLC, GCLM, and NQO1 were slightly downregulated by exposing cells to H_2_O_2_, which was significantly restrained with SFN. These results are similar to previous findings, showing that NFE2L2 has the potential to be a valuable target protein for SFN in the antioxidant effect.

AMPK is an energy sensor for cellular energy homeostasis, affecting the cellular redox state by upregulating antioxidant enzymes [25,26]. It has been reported that phosphorylation of AMPK could lead to NFE2L2 nuclear translocation [27]. In a recent study, reducing ROS production was connected with AMPK-mediated NFE2L2 activation in mouse MECs [28]. Similarly, we further confirmed that AMPK activation is responsible for SFN-induced NFE2L2 nuclear accumulation, and the mechanism is proved to play a crucial role in the suppression of ROS production. These results demonstrate that activating NFE2L2 represents a mechanism for AMPK to regulate antioxidant responses and that activation of AMPK may represent therapeutic targets for suppressing oxidative damage.

The imbalance between ROS production and antioxidant defense systems is the early cause of GMECs injury that may subsequently result in epithelium dysfunction. Numerous studies have shown that oxidative stress elicited by H_2_O_2_ can cause apoptosis [29]. The mitochondrial apoptotic pathway can be triggered by ROS, leading to caspase-3 activation, along with a change in the Bax/BCL-2 ratios [30]. In this study, SFN, in a dose-dependent manner, not only mitigated H_2_O_2_-induced apoptosis by a decrease in apoptotic rate, caspase-3, and Bax expression but also upregulated the expression of antiapoptotic BCL-2 in GMECs. These results strongly highlight the antiapoptotic potential of SFN. Indeed, coupled with the failure of SFN to decrease H_2_O_2_-induced ROS production and apoptosis in NFE2L2-silenced GMECs, the antioxidant effects of SFN were largely abolished when NFE2L2 expression was depleted. Thus, we speculate that activating NFE2L2 in the mammary gland represents a mechanism for SFN to alleviate oxidative damage.

## 4. Materials and Methods

### 4.1. Regents

Sulforaphane (purity > 99%, HY-13755) was obtained from MedChemExpress. H_2_O_2_ solution (Sigma, 323381) was bought from Sigma Aldrich (USA). Compound C (AbMole, M2238) was purchased from AbMole BioScience (Houston, TX, USA).

### 4.2. Cell Culture and Identification

Three clinically healthy Guanzhong dairy goats (3 years old; 70 to 90 days postpartum) obtained from a commercial dairy herd in Shaanxi Province (P. R. China) were selected for the study. The goats were pathogen-free via three consecutive bacteriologic determinations 14 days before the start of the experiment. The animal procedures were completed in strict accordance with the Guidelines for the Care and Use of Laboratory Animals and were approved by the Institutional Animal Care and Use Committees at Northwest A&F University.

Primary GMECs were prepared from 3 lactating dairy goats according to the previous method [31] with slight modifications. Briefly, mammary tissue pieces (0.5 to 1 mm^3^) were transferred into 24-well cell culture plate (Thermo Fisher Scientific, Waltham, MA, USA, 142485) and cultured in Dulbecco Modified Eagle Medium/nutrient mixture Ham F-12 (DMEM/F12; Thermo Fisher Scientific, Waltham, MA, USA, 12500) supplemented with 10% fetal bovine serum (FBS; Thermo Fisher Scientific, Waltham, MA, USA, 10270) and 1% penicillin/streptomycin (Solarbio, Beijing, China, P1400) at 37 °C in a humidified atmosphere with 5% CO_2_. The medium was replaced every 48 h until the cells migrated out of the tissue. The isolated cells attached to the dish surface were passaged by digestion with 0.25% trypsin/ethylenediaminetetraacetic acid (EDTA). Pure GMECs were selected after 2 passages and used between passages 3 and 7 for the experiments.

### 4.3. Cell Toxicity Assay

The cytotoxicity of SFN on GMECs was measured using Cell Counting Kit 8 (CCK 8, AbMole Bioscience Inc., Houston, TX, USA). In brief, the primary GMECs were seeded in 96-well plates (8 × 10^3^ cells/well) and treated with various concentrations of SFN and/or H_2_O_2_. After cultivating for different times, cells were treated with 10 μL CCK 8 solution and incubated for 2 h at 37 °C. The absorbance of the samples was determined at 450 nm.

### 4.4. Analysis of ROS

Intracellular ROS generation was detected using 2,7-dichlorodihydrofluorescein diacetate (DCFH-DA, Beyotime, Shanghai, China, S0033M) fluorescent dye. In brief, GMECs were subjected to different treatments and incubated with 10 μM DCFH-DA in serum-free culture media at 37 °C for 20 min. Following three washes with PBS, images were captured with a fluorescence microscope (Olympus, Tokyo, Japan). Mean fluorescence intensities of images were quantified using Image J software (1.53a/Java 1.8.0_112, NIH, USA) [32].

### 4.5. Determination of SOD, MDA, and GSH/GSSH

After cell lysis and centrifugation, the supernatants were collected for subsequent tests. The SOD activity, MDA production, and the ratio of GSH/GSSG were measured according to manufacturers’ instructions for detection kits (Beyotime, Shanghai, China) as previously described [33].

### 4.6. Apoptosis Analysis

Apoptosis assay was performed using an annexin V/PI apoptosis detection kit (Beyotime, Shanghai, China). The cells were harvested for annexin V/PI staining according to the manufacturer’s instructions and analyzed using flow cytometry (BD FACSAria III, BD Biosciences, Franklin Lakes, NJ, USA). Terminal deoxynucleotidyl transferase-mediated dUTP nick-end labeling (TUNEL) kit (Beyotime, Shanghai, China) was applied to further determine cell apoptosis according to the manufacturer’s protocol. GMECs were fixed with 4% paraformaldehyde for 30 min and incubated in 0.3% Triton X-100 for 5 min at room temperature, followed by treatment with TUNEL reaction mixture for 60 min at 37 ℃. After three washes with PBS, the images were acquired using fluorescence microscope (Olympus, Tokyo, Japan).

### 4.7. Quantitative Real-Time PCR Analysis

Total RNA was extracted using RNAiso Plus (TaKaRa, Dalian, China). cDNA was synthesized using the PrimeScript RT Master Mix reverse transcription kit (TaKaRa, Dalian, China) following the manufacturer’s protocol. Quantitative real-time PCR (qRT-PCR) was conducted using an SYBR Green qPCR master mix (Takara, Dalian, China) under the following parameters: 95 ℃ for 30 s, 40 cycles of 95 °C for 5 s, and 60 °C for 30 s, followed by a melting curve. The primers for the qRT-PCR are listed in Table 1. GAPDH mRNA was used as an internal reference [34], and 2^−ΔΔCt^ method was used to analyze relative mRNA levels.

### 4.8. Western Blot Analysis

Western blot was performed as described previously [35]. GMECs were lysed with ice-cold RIPA buffer (Solarbio, Beijing, China) containing protease and phosphatase inhibitor cocktail (Solarbio, Beijing, China). The protein concentrations were examined with a BCA kit (Epizyme, Shanghai, China). The quantified protein from each group was separated by 10% sodium dodecyl sulfate–polyacrylamide gel electrophoresis (SDS-PAGE) and transferred to polyvinylidene difluoride membranes (Millipore, Bedford, MA, USA). Following blocking with 5% skimmed milk in Tris-Buffered Saline and Tween 20 (TBST) buffer for 2 h at room temperature, the membranes were incubated with primary antibodies at 4 ℃ overnight. After washing five times with TBST for 5 min each time, the membrane was incubated with horseradish peroxidase-conjugated secondary antibodies for 2 h at room temperature. The blots were washed three times with TBST and determined with a chemiluminescence detection system (Bio-Rad Laboratories, Hercules, CA, USA). The levels of target protein bands were quantified with Image J software. The primary antibodies used for Western blot are listed in Table 2.

### 4.9. Immunofluorescence Staining

GMECs were incubated on coverslips and subjected to indicated treatments. Cells were fixed with 4% paraformaldehyde, permeabilized with 0.1% Triton X-100, and blocked with 10% FBS in PBS for 1 h. After overnight incubation at 4 °C with the NFE2L2 primary antibody (1:400, Abcam, Cambridge, MA, USA, ab137550), cells were treated with the secondary antibody for 1.5 h at room temperature. Then, DAPI was used to stain nuclei. The cells were imaged using confocal microscope (Nikon A1Rsi, Tokyo, Japan).

### 4.10. RNA Interference

The transfection of small interfering RNA (siRNA) into GMECs was carried out using Lipofectamine 3000 transfection reagent (Invitrogen, Carlsbad, CA, USA). Briefly, siRNA and Lipofectamine 3000 reagent were mixed in Opti-MEM Reduced Serum Media (Invitrogen, Carlsbad, CA, USA) and incubated for 15 min at room temperature. After washing the cells with Opti-MEM medium, the mixtures were added (Invitrogen, Carlsbad, CA, USA). Subsequently, cells were harvested 48 h after transfection, and further experiments were performed. The negative control siRNA (si-NC) and NFE2L2 siRNA (si-NFE2L2) target sequences were designed and synthesized by GenePharma (Shanghai, China), as shown in Table 3.

### 4.11. Statistical Analysis

Data are presented as means ± SEMs. Statistical analysis was performed using GraphPad Prism 8.0.1 (GraphPad, San Diego, CA, USA). Differences for experiments among three or more groups were determined by one- or two-way analysis of variance (ANOVA) followed by Tukey or Dunnett’s post hoc tests. Results of statistically significant differences are indicated by asterisks (* *p* < 0.05, ** *p* < 0.01, *** *p* < 0.001, **** *p* < 0.0001).

## 5. Conclusions

Our findings clearly indicate that SFN suppresses H_2_O_2_-induced oxidative damage in primary goat mammary epithelial cells. Furthermore, SFN decreased ROS production, enhanced antioxidant capacity, and reduced apoptosis. The underlying mechanism is that SFN activated the AMPK/NFE2L2 signaling pathway and inhibited the caspase-dependent pathway of apoptosis. This study provides the basis for SFN to prevent oxidative stress often seen in high-yielding dairy goats, which will further improve the accuracy of pharmacology and mechanism-based SFN repurposing. Thus, the clinical application of SFN for its protective effect on oxidative stress in high-yielding dairy animals warrants further study.

## Figures and Tables

**Table 1:** Primer sequences for RT-qPCR.

Gene	Sequence (5′ to 3′)	Length, bp
*NFE2L2*	F: CCAACTACTCCCAGGTAGCCC	227
	R: AGCAGTGGCAACCTGAACG	
*HMOX1*	F: CAAGCGCTATGTTCAGCGAC	206
	R: GCTTGAACTTGGTGGCACTG	
*NQO1*	F: ACTGTGTCGGACCTGTATGC	363
	R: CAGAGAGTACATGGAGCCGC	
*GCLM*	F: AATCTTGCCTCCTGCTGTGTGATG	138
	R: GATGCTCTCCTGAAGTGCTTCTTGG	
*GCLC*	F: CATTTGCAAAGGTGGCAACGC	301
	R: CTGCTTGTAGTCGGGATGCT	
*GAPDH*	F: ACCTGCCAAGTATGATGAG	118
	R: AGTGTCGCTGTTGAAGTC	

**Table 2:** Antibody information for Western blot.

Name of Antibody	Dilution Ratio	Article Number	Manufacturer
Bax	1:2000	50599-2-lg	Proteintech (Wuhan, China)
Bcl2	1:1000	12789-1-AP	Proteintech (Wuhan, China)
Caspase 3	1:1000	14220	Cell Signaling Technology (Boston, MA, USA)
NFE2L2	1:2000	ab137550	Abcam (Cambridge, USA)
HMOX1	1:2000	10701-1-AP	Proteintech (Wuhan, China)
NQO1	1:2000	11451-1-AP	Proteintech (Wuhan, China)
GCLC	1:2000	12601-1-AP	Proteintech (Wuhan, China)
GCLM	1:2000	14241-1-AP	Proteintech (Wuhan, China)
AMPK	1:1000	5831	Cell Signaling Technology (Boston, MA, USA)
p-AMPK	1:1000	2535	Cell Signaling Technology (Boston, MA, USA)

**Table 3:** Oligos for siRNA.

Gene	Sequence (5′ to 3′)
*si-NC*	UUCUCCGAACGUGUCACGUTT ACGUGACACGUUCGGAGAATT
*si-NFE2L2-1*	GAGGCCAGAUAUUAAGAAATT UUUCUUAAUAUCUGGCCUCTT
*si-NFE2L2-2*	CCGGUUGACAGUGAAUUCATT UGAAUUCACUGUCAACCGGTT
*si-NFE2L2-3*	GGUAGCCACUGCUGAUUUATT UAAAUCAGCAGUGGCUACCTT

## Data Availability

Due to data confidentiality requirements, the manuscript cannot provide raw data.
